# Peanut Shell Valorization: Effects of Particle Size on Techno‐Functional Attributes, Mineral Profile, Starch Digestibility, and Bioactive Properties of Cookies

**DOI:** 10.1111/1750-3841.71145

**Published:** 2026-06-09

**Authors:** Ülgen İlknur Konak Alkış, Fundagül Erem, Nazlı Şahin

**Affiliations:** ^1^ Department of Food Engineering Faculty of Engineering Avrasya University Trabzon Türkiye; ^2^ Department of Food Engineering Faculty of Engineering Ankara University Ankara Türkiye; ^3^ Department of Food Engineering Faculty of Engineering Zonguldak Bülent Ecevit University Zonguldak Türkiye; ^4^ Department of Food Engineering Faculty of Engineering Karamanoğlu Mehmetbey University Karaman Türkiye

## Abstract

Peanuts are among the most widely produced nuts globally, generating greater quantities of shell residues than other nut by‐products, thereby representing a highly abundant source of lignocellulosic fiber and phenolic compounds. This study examined the effect of peanut shell ground to different sizes (212, 500, and 800 µm) on the physical, chemical, techno‐functional, and textural properties of cookies. As the particle size decreased, the dough's hardness increased, while its stickiness and strength decreased (*p* < 0.05). Adding peanut shell to cookies resulted in approximately a 100‐fold increase in crude fiber content, a 1.8‐fold increase in total phenolic content, and a 2.4‐fold increase in antioxidant activity compared to the control sample (*p* < 0.05). Moreover, as the particle size of the peanut shell decreased from 800 to 212 µm, starch digestibility declined, and the mineral content increased in the cookies (*p* < 0.05). Incorporating peanut shell powder into cookies reduced their thickness by 8%–15% but increased the spread ratio by 18%–33% compared to the control cookies (*p* < 0.05). The cookies' hardness and fracturability decreased by about 27%–47% and 1%–5%, respectively, as the particle size of the peanut shell powder increased (*p* < 0.05). These findings suggest that peanut shell could serve as an important source of fiber and phenolic compounds for functional cookie production.

**Practical Applications:**

Peanut shells, often discarded as processing waste, can be transformed into a valuable ingredient for healthier cookies. Adding peanut shell powder significantly increases fiber, antioxidants, and minerals while reducing starch digestibility. Using peanut shells in bakery products not only supports sustainable waste utilization but also offers an affordable way to create functional foods with added health benefits, an opportunity for both industry innovation and improved public nutrition. The significant increase in dietary fiber and antioxidant activity suggests that its incorporation could support clean‐label product development and align with consumer demand for health‐oriented food products.

## 1 Introduction

The Food and Agriculture Organization reported that global peanut production reached around 54 million tons in 2023 (FAOSTAT 2025). It is believed that peanuts, commonly called goober or groundnuts, originated in Central and South America. Today, more than 300 varieties of peanuts are grown worldwide, including in China, India, Africa, Japan, South America, and the United States. The production of peanuts generates agricultural residues and by‐products such as peanut meal, skin, vine, and hull (Toomer 2020). Peanut shells, also known as hulls, account for approximately 20% of the peanut's weight. They have a fibrous, lignocellulosic structure comprising 45% cellulose, 6% hemicellulose, and 36% lignin (Pączkowski et al. 2021). Lignocellulosic biomass, predominantly made up of cellulose, hemicellulose, and lignin, is considered a potential ingredient for various industries such as food, cosmetics, and pharmaceuticals due to its bioactive properties (Bhatia et al. 2019). Recently, interest in lignin has increased because of its functional and bioactive properties, which could make it a valuable food ingredient (Gil‐Chávez et al. 2021; Tao et al. 2020) and food packaging agent (Anushikha and Gaikwad 2023). Cellulose and its derivatives are widely used in different applications, including as emulsion and foam stabilizers, fat replacers, edible coating materials, and encapsulation agents. In addition, cellulose has health benefits linked to hypoglycemic activity, hypolipidemic activity, and support for intestinal system function (He et al. 2021).

Besides their fiber qualities, the antioxidant properties of agricultural wastes enhance their potential uses. Specifically, plant cell wall polysaccharides attract attention as natural, inexpensive, and potentially bioactive carbohydrates (Granato et al. 2022). Polyphenolic compounds, known for their antioxidant and antimicrobial effects, are among the main components of dietary fiber alongside cell wall polysaccharides, and these two are reported to potentially interact with each other. Although the exact mechanism is not fully understood yet, it is believed that while polyphenols may act as natural preservatives in foods, cell wall components might facilitate the controlled release of polyphenols in the human digestive system by protecting them (Siemińska‐Kuczer et al. 2022; X. Liu et al. 2020). Phenolic compounds in peanut shells are primarily responsible for their antioxidant activity (Adhikari et al. 2019). Comparative studies have demonstrated that peanut shells contain high levels of phenolic compounds (≈240 mg GAE/g extract) compared to hazelnut shells (≈160 mg GAE/g extract) and almond shells (≈22 mg GAE/g extract), although lower than walnut shells (≈328 mg GAE/g extract). Similarly, antioxidant activity assessed by DPPH assay indicated that peanut shells (IC50: 67.72) had higher antioxidant activity than hazelnut shells (IC50: 82.11) and almond shells (IC50: 900.9), although lower than walnut shells (IC50: 18.33). The same trend was also observed in the ABTS and FRAP assays (Salem et al. 2022). In addition, peanut shells were found to have potent antibacterial activity against harmful bacteria by disrupting plasma membrane permeability and DNA content (Al‐Hazmi and Naguib 2023). Besides its beneficial properties, the use of lignocellulosic biomass as a food ingredient may raise food safety concerns, particularly in industrial applications where large‐scale handling and processing are involved. Although safety studies specifically focusing on peanut shells are limited, it can be assumed that peanut shells are exposed to similar environmental and post‐harvest contaminants as the kernels inside. Therefore, the risks identified for the peanut kernels are also valid for the peanut shells. Peanuts are susceptible to mycotoxin contamination, especially to aflatoxin (Melo et al. 2025; Meneely et al. 2023), which primarily affects the kernels. In addition, the heavy metal content of soil has been reported to influence contaminant levels in peanuts (Zhang et al. 2024). Post‐harvest cross‐contamination of pathogens has also been reported in peanuts (Chang et al. 2013), indicating that pathogen contamination may be more prevalent in the peanut shell. Therefore, comprehensive safety assessments are essential before peanut shell‐derived ingredients can be considered for human consumption.

Agricultural wastes can serve as important sources of valuable functional compounds that can be recovered and reused in food products. Peanuts' waste contains many functional compounds such as protein, fiber, and polyphenols, and can be added as functional ingredients in processed foods. Among these wastes, peanut skin or its extract is commonly used in cereal‐based foods such as noodles (Incedayı and Türkmen Erol 2021) and cookies (De Camargo et al. 2014). However, to our knowledge, no studies have been conducted on the valorization of peanut shells as an ingredient in cereal‐based products. As mentioned before, peanut shell is a lignocellulosic by‐product characterized by high cellulose and lignin content, which structurally and functionally distinguishes it from conventional cereal brans that are predominantly composed of polysaccharides such as arabinoxylans and β‐glucans. These compositional differences may lead to distinct technological behaviors when incorporated into bakery formulations. Therefore, this study aims to investigate the effect of peanut shell powders (PSPs) with different particle sizes on the physicochemical and technological properties of cookies.

## 2 Materials and Methods

### 2.1 Materials

Peanut (*Arachis hypogaea* L.) shells were kindly donated by a local peanut facility in Osmaniye, Türkiye, in January 2023. Flour (10.16% protein, 2.19% fat, 0.64% ash, Sinangil, Türkiye), castor sugar (Dr. Oetker, Türkiye), margarine (79% of fat, Sana, Türkiye), and baking powder (Dr. Oetker, Türkiye) were purchased from a local market. All the chemicals used were of analytical grade (Sigma‐Aldrich, Germany).

### 2.2 Methods

#### 2.2.1 Preparation of Peanut Shell

The peanut shells were supplied by a peanut processing facility and provided in a clean, ready‐to‐use state. The PS was ground into powder with a coffee grinder (Russell Hobbs, 23120–56, China) and divided into portions of 100 g. Then, each portion was shaken with a sieving machine (Çeliktest, ÇMS11‐011, Türkiye) by using different aperture sizes of sieves (1000, 800, 500, and 212 µm) for 5 min. The materials that remained on each sieve were weighed, and the yield was calculated as a percentage by dividing the mass of the material on each sieve by the mass of the total amount obtained. The remaining samples on each sieve were labeled as PSP10, PSP8, PSP5, and PSP2 for the sieves with openings of 1000, 800, 500, and 212 µm, respectively. The sample that passed through the sieve of 212 µm was labeled as PSP < 2.

#### 2.2.2 Thermo‐Mechanical Properties of Wheat Flour and PSP

The Mixolab (Choppin Technologies, France) was used to analyze the thermo‐mechanical behaviors of dough samples made with 50 g of WF or WF‐PSP blends. The standard Chopin+ protocol was performed as follows: Mixing speed at 80 min^−1^, initial temperature at 30°C for 8 min, heating to 90°C (at a rate of 4°C/min), holding at 90°C for 7 min, cooling to 50°C (at a rate of 4°C/min), and holding at 50°C for 5 min. All analyses were performed at a constant water absorption level of 57.3%, determined from preliminary analysis of wheat flour (WF), to achieve a target dough consistency of approximately 1.1 ± 0.05 Nm. The hydration level was subsequently applied to all WF‐PSP blends to enable direct comparison of thermo‐mechanical properties under constant water conditions. The process was repeated twice, and the dough stability, starch gelatinization speed, retrogradation index, and viscosity index were evaluated (ICC 2006).

#### 2.2.3 Water and Oil Absorption Capacities of WF and PSP

The method of Marchetti et al. (2018) was performed to determine the water absorption capacity (WAC) and oil absorption capacity (OAC) of all PSP samples and WF. Briefly, 1 g of the sample was mixed with 10 mL of water or sunflower oil. Then, the mixture was centrifuged (Nuve NF 800R, Ankara, Türkiye) at 3000 × *g* for 20 min after holding at 25 ± 1°C for 30 min. The pellets were weighed, and the results were expressed as g of water or oil per g of dry weight of sample. Experiments were conducted in quadruplicate.

#### 2.2.4 Chemical Analyses of WF and PSP

The moisture (934‐01), ash (923‐03), protein (960‐52), and lipid (920‐39) content of WF and PSP samples were determined according to AOAC (2000) in triplicate. ICC's method was used for crude fiber analysis (ICC 1972). The neutral detergent (NDF), acid detergent (ADF), and acid detergent lignin (ADL) contents were determined with an Ankom Fibre Analyzer (Model 200, Ankom Technology, NY) according to the Ankom Operator's Manual. Cellulose content was calculated by subtracting the amount of ADL from ADF. Hemicellulose content was determined as the difference between NDF and ADF (Dziedzic et al. 2018). The mineral content was determined using inductively coupled plasma–optical emission spectroscopy (ICP‐OES, Vista series). Dried samples (0.2 g) were subjected to closed‐vessel microwave digestion using a MARS 5 system (CEM Corporation, Stallings, NC, USA) with a mixture of 7 mL HNO_3_, 1 mL HCl, and 1 mL H_2_O_2_. The digestion program consisted of a ramp to 100°C over 10 min followed by a 10 min holding period, and then a ramp to 180°C over 10 min with an additional 10 min holding time. After cooling, the digests were diluted to volume with deionized water prior to ICP‐OES analysis. Mineral concentrations were determined using ICP‐OES (Vista series, Agilent Technologies, USA). Calibration was performed using certified multi‐element standard solutions (0–10 mg/L; Certipur, Merck, Darmstadt, Germany), with correlation coefficients (*R* ^2^) > 0.999. Quality control included reagent blanks and triplicate analysis of each sample (Skujins 1998).

The extraction of samples for total phenolic content (TPC) and antioxidant activity (AA) analyses was performed according to the method of Meng et al. (2020) with slight modifications. Samples (1 g) mixed with 20 mL of 80% methanol were shaken at room temperature for 3 h, then centrifuged (Nuve NF 800R, Ankara, Türkiye) at 4100 rpm for 30 min. The supernatants were filtered through 4–12 µm pore‐sized filter papers and maintained at −20°C until analysis (within 1–2 days) to minimize oxidative degradation. TPC was determined with Folin–Ciocalteu's phenol reagent method by measuring the absorbance with a spectrophotometer (Shimadzu UV‐1800, Kyoto, Japan) at 750 nm (Ramírez‐Maganda et al. 2015). The results were expressed as mg gallic acid equivalent per gram dry weight of sample by using a calibration curve (*y* = 5.218*x* − 0.036, *R* ^2^ = 0.997) obtained with gallic acid (35–65 mg/L). The method of Meng et al. (2020) was slightly modified to perform antioxidant activity analysis. The radical scavenging activity of the sample extracts was determined by mixing 1 mL of sample extract with 4 mL of 75 µM 1,1‐diphenyl‐2‐picryl hydrazyl (DPPH) and measuring the absorbance with a spectrophotometer at 516 nm after incubating it at room temperature in the dark for 30 min. Trolox (10–140 µM) was used to generate a calibration curve (*y* = 0.558*x* − 0.710, *R* ^2^ = 1.000) and the results were given as mg Trolox equivalent per gram dry weight of sample. TPC and DPPH analysis were performed in quadruplicate.

#### 2.2.5 Preparation of Cookies

The control was produced with 100 g WF, 50 g castor sugar, 38 g margarine, 0.5 g baking powder, and 20 mL water. WF was replaced with PSPs (PSP8, PSP5, and PSP2) at a concentration of 15% on a weight basis. Our preliminary studies showed that a substitution level of more than 15%, especially for larger particle sizes, adversely affected the sensory and textural properties of the cookies. In addition, a higher substitution level (> 15%) resulted in noticeable deterioration in dough handling properties. The fat and sugar were creamed in a mixer (Prochef Xl, Schafer, Germany) at medium speed for 4 min. After that, the WF or WF‐PSP blends and baking powder were added and mixed for 4 min. Water (55°C) was added to the mixture and mixed for 2 min. The water was used at 55°C to facilitate the homogeneous dispersion of margarine and dissolution of sugar in the dough, thereby improving dough handling properties. The dough was manually sheeted to a thickness of 4 mm, and then the sheet was cut to 4.5 cm in diameter with a die. Cookies were baked in a preheated oven at 180°C for 20 min and allowed to cool to 25 ± 1°C (Agrahar‐Murugkar et al. 2015). Cookies containing PSP8, PSP5, and PSP2 were coded as CS8, CS5, and CS2, respectively.

#### 2.2.6 Texture Analysis of Cookie Dough

The texture characteristics of cookie dough (4 dough for each formulation in each batch) were measured using a TA.XT Plus (Stable Micro Systems, UK) equipped with a 5 kg load cell. The dough was allowed to rest for 15 min in a 50 mL glass beaker. A penetration test was conducted with a cylindrical probe (2 mm in diameter) at a distance of 10 mm and a test speed of 0.5 mm/s to measure the dough firmness (*N*) (Raymundo et al. 2014). Stickiness (*N*) and strength/cohesiveness (mm) of dough samples were measured with a Chen–Hoseney Dough Stickiness Rig (A/DSC). The test speed was 0.5 mm/s and the trigger force was 40 g (Jin et al. 2020). The measurements were replicated eight times. Dough samples containing PSP8, PSP5, and PSP2 were coded as DS8, DS5, and DS2, respectively.

#### 2.2.7 Chemical Analyses of Cookies

Proximate composition, mineral content, TPC, and AA of the cookies were determined according to the procedures given in the section for chemical analysis of WF and PSP samples in triplicate. The crude fiber content of the cookies was analyzed (ICC 1972) after removing the lipids by petroleum ether extraction. In vitro starch digestibility of cookies—including rapidly digestible starch (RDS), slowly digestible starch (SDS), total digestible starch (TDS), and resistant starch (RS)—was determined using the digestible starch assay kit (K‐DSTRS; Megazyme), following the enzymatic procedure described by McCleary and Monaghan (2002). Samples were enzymatically hydrolyzed using pancreatic α‐amylase (PAA) and amyloglucosidase (AMG) in 50 mM maleate buffer (pH 6.0) at 37°C for up to 4 h. Aliquots were taken at 20 min (RDS), 120 min (SDS: starch at 120 min—starch at 20 min), and 240 min (TDS and RS). The reaction was terminated by transferring 1.0 mL aliquots into 20 mL of 50 mM acetic acid (pH 4.5). Residual maltose was hydrolyzed with AMG (100 U/mL), and glucose was quantified using the GOPOD reagent at 510 nm. RS was determined by centrifugation, followed by washing with 50% v/v aqueous ethanol and resuspension in cold 1.7 M sodium hydroxide to dissolve the RS. The solution was then neutralized and hydrolyzed with AMG, followed by glucose measurement at 510 nm using the GOPOD reagent. All starch fractions (RDS, SDS, TDS, and RS) were expressed as a percentage of dry weight (% dw) based on moisture‐corrected values.

#### 2.2.8 Color Measurement of Cookies

Color measurements (L*, a*, and b*) were made on four points of the top surface of three cookies in each batch using a spectral colorimeter (CHN CS‐410, Hangzhou, China) with a D65 illuminant and 10° observer angle and CIE Lab scale. The total color difference (∆*E*) was calculated using Equation 1:

(1) $$\Delta E=\sqrt{{\left(L_{0}^{*}-\;L^{*}\right)}^{2}+{\left(a_{0}^{*}-a^{*}\right)}^{2}+{\left(b_{0}^{*}-b^{*}\right)}^{2}}$$

where $L_{0}^{*}$, $a_{0}^{*}$, and $b_{0}^{*}$ are the color values of the control cookies, $\;L^{*}$, $a^{*},$ and $b^{*}$ are the color values of the PSP‐containing cookies.

#### 2.2.9 Spread Ratio of Cookies

The diameter and thickness of 10 cookies for each formulation in each baking run were measured using a digital Vernier caliper. The spread ratio was calculated by dividing the average value of the diameter by the average value of the thickness of the cookies (AACC 2010).

#### 2.2.10 Texture Analysis of Cookies

The hardness (*N*) and fracturability (mm) of 8 cookies for each formulation in each baking run were measured using a TA.XT Plus (Stable Micro Systems, UK) equipped with a 3‐Point Bending Rig (HDP/3 PB) and a 5 kg load cell. The test parameters were as follows: Distance of 10 mm, trigger force of 50 g, pre‐test speed of 1.0 mm/s, test speed of 3.0 mm/s, and post‐test speed of 10.0 mm/s (Altındağ et al. 2015). The measurements were replicated eight times.

#### 2.2.11 Statistical Analysis

Significant differences between the samples were determined by performing a one‐way analysis of variance (ANOVA) using SAS System Software (SAS OnDemand for Academics). Significant parameters were evaluated by Duncan's multiple range test at the 95% confidence level. Dough preparation and cookie baking were performed in two separate replications. At least duplicate analysis was performed in each replicate. Analyses carried out with different replicates were specifically indicated in the individual methods. The results were given as mean ± standard deviation.

## 3 Results and Discussion

### 3.1 Properties of PSP and WF

Proximate composition, WAC, and OAC of WF and PSP samples, as well as the percentage yield of PSP fractions, are given in Table 1. Smaller particle sizes led to increased protein, lipid, and ash contents in PSP fractions, particularly in PSP2 and PSP < 2, whereas crude fiber contents decreased. Similar results were observed in cocoa bean shells, where finer particles had higher fat and protein levels; this was attributed to increased extraction efficiency due to larger surface area (Botella‐Martínez et al. 2021). Furthermore, ash tends to concentrate in finer‐sized fractions during biomass processing because inorganic materials are small and brittle (Queirós et al. 2020). Compared to this study, Imran et al. (2022) determined higher protein, crude fiber, and ash content but lower lipid values for the unfractionated peanut shell samples.

**TABLE 1:** Physical and chemical characteristics of wheat flour and different particle‐sized peanut shell powders.

	WF	PSP	PSP10	PSP8	PSP5	PSP2	PSP<2
Moisture	9.97^a^ ± 0.05	8.10^b^ ± 0.08	7.53^c^ ± 0.23	7.88^b^ ± 0.23	7.97^b^ ± 0.10	7.25^b^ ± 0.13	5.76^d^ ± 0.11
Protein (% dw)	10.16^a^ ± 0.08	2.94^d^ ± 0.09	2.00^f^ ± 0.09	2.16^e^ ± 0.04	2.16^e^ ± 0.04	4.41^c^ ± 0.04	6.85^b^ ± 0.05
Lipid (% dw)	2.19^b^ ± 0.24	1.79^c^ ± 0.21	0.36^f^ ± 0.04	0.93^e^ ± 0.03	0.88^e^ ± 0.05	1.48^d^ ± 0.13	3.55^a^ ± 0.05
Ash (% dw)	0.64^e^ ± 0.02	1.73^c^ ± 0.01	1.49^d^ ± 0.02	1.45^d^ ± 0.11	1.46^d^ ± 0.04	2.44^b^ ± 0.05	3.81^a^ ± 0.06
Crude fiber (% dw)	0.21^e^ ± 0.01	40.02^b^ ± 0.83	43.25^a^ ± 0.95	43.50^a^ ± 0.17	42.97^a^ ± 0.17	36.73^c^ ± 0.35	30.95^d^ ± 0.45
OAC (g/g dw of sample)	0.84^f^ ± 0.01	2.45^c^ ± 0.02	2.49^c^ ± 0.04	2.16^d^ ± 0.06	2.07^e^ ± 0.05	3.39^b^ ± 0.05	3.88^a^ ± 0.04
WAC (g/g dw of sample)	0.76^e^ ± 0.01	4.89^c^ ± 0.19	4.93^c^ ± 0.17	4.50^d^ ± 0.24	4.60^d^ ± 0.06	6.16^b^ ± 0.06	6.84^a^ ± 0.24
Yield (%)	—	—	18.18^c^ ± 1.04	22.60^b^ ± 1.40	32.42^a^ ± 0.87	21.76^b^ ± 0.88	5.05^d^ ± 1.88

*Note*: Different superscript lowercase letters in the same row represent significant differences (*p* < 0.05). PSP10, PSP8, PSP5, and PSP2 are the PSP portions that remain on a sieve with an aperture size of 1000, 800, 500, and 212 µm, respectively, while PSP < 2 is the portion that passed through the sieve with an aperture size of 212 µm.

Abbreviations: PSP, peanut shell powder; WF, wheat flour.

The WAC and OAC values were found significantly higher (*p* < 0.05) for the smaller particle‐sized PSP fractions (Table 1) due to the higher surface area of smaller particles and increased hydration rate (Drakos et al. 2017) and probably the higher protein content (Olakanmi et al. 2024). The WAC and OAC values obtained for the PSP sample were higher than the findings of L. Wang et al. (2023). The high OAC and WAC are indicators of the presence of higher amounts of apolar amino acids and hydrophilic carbohydrates in the flour (Akpapunam and Darbe 1994). The water absorption of flour is affected by the protein content, the amount of damaged starch, and the presence of non‐starch carbohydrates (Ma et al. 2007).

According to the sieve analysis results, the highest yield was obtained from PSP5, followed by PSP8 and PSP2, respectively. The yield values for the other sieves were found to be below 20% (Table 1). Therefore, for cookie production, the process continued with PSP2, PSP5, and PSP8. NDF, ADF, and ADL contents of selected PSP fractions were determined and are shown in Table 2. The highest values were observed for PSP5, and the differences between the samples were statistically significant (*p* < 0.05). PSP2 exhibited substantially (*p* < 0.05) lower cellulose content than PSP8 and PSP5, while hemicellulose content was highest in PSP5, followed by PSP2 and PSP8. Rico et al. (2018) reported higher lignin and hemicellulose but lower cellulose contents for peanut shell than those found in this study (Table 2). The lignin content of PSP fractions aligned with the results reported by Husna and Vasantharuba (2022), however, they observed higher hemicellulose and lower cellulose content compared to our findings. Milling is a fractionation‐driven, non‐linear process governed by anisotropic fracture of heterogeneous lignocellulosic structures (Oyedeji et al. 2020). Intermediate particle sizes may represent an optimal fracture zone where lignified and hemicellulose‐rich regions are preferentially separated from more amorphous components, resulting in their relative enrichment in specific fractions. Zhu et al. (2010) and Speroni et al. (2020) also demonstrated lower insoluble fiber content after ultrafine grinding of wheat bran dietary fiber and micronization of olive pomace, respectively. It was reported that the increase in surface‐to‐volume ratio and cell wall disruption with the reduction in the particle size leads to an improvement in the accessibility of dietary components (Du et al. 2020).

**TABLE 2:** Mineral content and fiber analyses of peanut shell powder fractions.

	PSP8	PSP5	PSP2
NDF (%)	80.43^b^ ± 0.09	86.86^a^ ± 2.36	76.82^c^ ± 0.52
ADF (%)	72.12^b^ ± 0.19	74.02^a^ ± 0.35	67.10^c^ ± 0.21
ADL (%)	31.16^b^ ± 0.13	32.22^a^ ± 0.13	29.52^c^ ± 0.49
Cellulose (%)	40.96^a^ ± 0.05	41.80^a^ ± 0.48	37.58^b^ ± 0.70
Hemicellulose (%)	8.31^c^ ± 0.27	12.84^a^ ± 0.59	9.72^b^ ± 0.30
Ca (mg/100 g)	104.95^c^ ± 0.82	107.73^b^ ± 0.98	135.04^a^ ± 1.21
Cu (mg/100 g)	0.17 ± 0.01	0.20 ± 0.05	0.19 ± 0.01
Fe (mg/100 g)	1.62^c^ ± 0.02	3.42^b^ ± 0.04	8.20^a^ ± 0.10
K (mg/100 g)	281.15^b^ ± 2.12	285.83^b^ ± 97.79	610.64^a^ ± 56.50
Mg (mg/100 g)	10.28^c^ ± 0.09	14.57^b^ ± 0.16	25.02^a^ ± 0.26
Mn (mg/100 g)	0.23^c^ ± 0.00	0.26^b^ ± 0.00	0.33^a^ ± 0.01
P (mg/100 g)	50.46^a^ ± 2.42	40.10^b^ ± 4.70	51.80^a^ ± 2.35

*Note*: Different superscript lowercase letters in the same row represent significant differences (*p* < 0.05). PSP8, PSP5, and PSP2 are the PSP portions that remain on a sieve with an aperture size of 800, 500, and 212 µm, respectively.

Abbreviations: ADF, acid detergent fiber; ADL, acid detergent lignin; NDF, neutral detergent fiber.

PSPs with different particle sizes showed significant differences in mineral content (*p* < 0.05) (Table 2). The mineral values of WF were 10.71 mg/100 g for calcium (Ca), 0.11 mg/100 g for iron (Fe), 117.94 mg/100 g for potassium (K), 5.36 mg/100 g for magnesium (Mg), 41.07 mg/100 g for phosphorus (P), 0.08 mg/100 g for manganese (Mn), and 0.08 mg/100 g for copper (Cu). All PSP had notably higher levels of essential minerals compared to WF. Among the samples, PSP2 showed the highest mineral concentrations in terms of all minerals tested. This trend was consistently observed across all analyzed minerals, indicating that reducing particle size increases the measured mineral concentration in peanut shell samples. These findings are also consistent with the higher ash content previously observed in the peanut shell samples (Table 1). The variation in mineral content among particle sizes can be attributed to the heterogeneous distribution of inorganic components within the tissues. In lignocellulosic materials, milling or grinding can break down the complex lignin–carbohydrate matrix, facilitating the release of entrapped compounds, including minerals (Silva et al. 2012).

**FIGURE 1:**
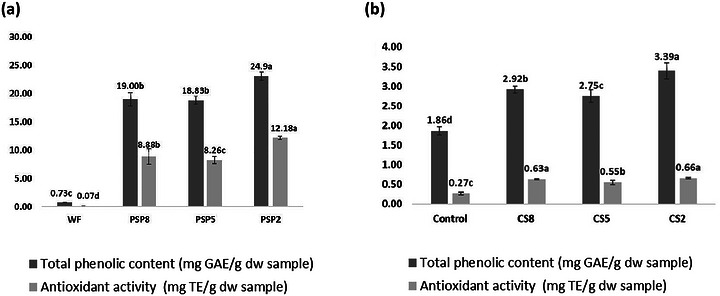
Total phenolic content and antioxidant activity values of (a) WF and different particle sizes of PSP, and; (b) cookie samples (different letters given with the mean values refer to significant differences among samples; *p* < 0.05).

There were substantial differences (*p* < 0.05) between the TPC and antioxidant capacity of WF and PSP samples (Figure 1a). PSP with the finest particles had the highest values, and compared to WF, the TPC and antioxidant capacity of PSP2 were approximately 40 times and more than 200 times higher, respectively. While there were no significant differences between the TPC values of PSP8 and PSP5, the antioxidant capacity of PSP8 was determined to be statistically higher than that of PSP5 (*p* < 0.05). Meng et al. (2020) and Adhikari et al. (2019) determined TPC values in the methanolic extracts of PS with conventional extraction as 3.62 mg GAE/g and 0.43–0.74 mg GAE/g, respectively. These values were quite low from the data obtained in this study (Figure 1a). On the other hand, the TPC value (27.6 mg GAE/g) found by Imran et al. (2022) was similar to PSP2, however, they determined higher antioxidant capacity values (44.6 mg TE/g) with the DPPH method. Depending on autohydrolysis at different temperatures, the antioxidant activity of PS with DPPH assay was determined in the range of 9.6‐11.7 mg TE/g (Rico et al. 2018), which was similar to the results found in this study (Figure 1a). Rico et al. (2018) showed that non‐isothermal aqueous treatment of PS resulted in the occurrence of soluble oligosaccharides and lignin‐derived substances, which exhibit high antioxidant activity. Finer particles (PSP2) provided a larger surface area and greater cell wall disruption, enhancing the release and extractability of phenolic compounds, thereby leading to significantly higher TPC and antioxidant capacity (Du et al. 2020).

### 3.2 Thermo‐Mechanical Properties

As dough characteristics, dough development time, stability, weakening of protein network (C2), starch gelatinization (C3), stability of the hot‐formed gel (C4), and starch retrogradation (C5) of the flour/blends were evaluated (Table 3). The C1 values of WF, WF‐PSP2, WF‐PSP5, and WF‐PSP8 were 1.14 ± 0.01, 1.16 ± 0.01, 1.01 ± 0.00, and 1.02 ± 0.00, respectively. The dough development time is the maximum time to reach 1.1 N.m torque and the stability revealed the resistance of the dough to prolonged shearing during mixing. The PSP blends showed lower stabilities but longer development times compared to WF. The increased development times in the PSP blends were likely due to the interference of PS particles with gluten formation. These fiber‐rich particles may inhibit flour protein aggregation, thereby prolonging the dough development process. Lower stabilities in PSP blends were primarily ascribed to the water competition action of shell polysaccharides in the dough system and the dilution of gluten protein. Similar results were reported by Xiong et al. (2017), who noted that bran addition in the bread dough system resulted in lower stability and longer development time. Moreover, the addition of PSP8 and PSP5 into WF significantly (*p* < 0.05) increased the development time of the dough compared to PSP2 (Table 3). This may be attributed to the fact that larger particles require more time to absorb water than finer particles, thereby resulting in longer development times. C3 represents the degree of starch gelatinization. There was no significant difference in C3 values between PSP8 and PSP5, and between PSP8 and PSP2. The result was similar to the report of T. Liu et al. (2016), who found that bran particle size did not show a significant impact on pasting properties. Furthermore, PSP addition resulted in reduced C5 values, which was the consequence of starch dilution. The addition of PSP disrupted the connections between amylose chains and limited the starch molecules' ability to crosslink when cooled, which possibly explains why PSP blends' retrogradation rates are lower than those of WF (Xiong et al. 2017). In addition, the C5–C4 value indicates starch retrogradation and the values were lower for PSP blends compared to the WF. This finding is consistent with the findings of Li et al. (2023), who found that water molecules are less available for starch retrogradation in whole WF due to the fibers' greater ability to absorb water than in white flour.

**TABLE 3:** Mixolab and texture parameters of dough.

Mixolab parameters of WF‐PSP blends	WF	WF/PSP8	WF/PSP5	WF/PSP2
Development time (min)	5.59^c^ ± 0.34	8.63^a^ ± 0.01	8.44^a^ ± 0.62	6.68^b^ ± 0.35
Dough stability (min)	10.01^a^ ± 0.11	9.77^ab^ ± 0.83	9.68^ab^ ± 0.22	9.27^b^ ± 0.12
C2 (N.m)	0.64^a^ ± 0.01	0.62^ab^ ± 0.02	0.57^b^ ± 0.01	0.59^b^ ± 0.01
C3 (N.m)	1.97^ab^ ± 0.08	1.99^ab^ ± 0.02	1.93^b^ ± 0.04	2.09^a^ ± 0.02
C4 (N.m)	1.61^a^ ± 0.08	1.36^b^ ± 0.09	1.33^b^ ± 0.01	1.43^b^ ± 0.05
C5 (N.m)	2.26^a^ ± 0.27	1.83^b^ ± 0.06	1.75^b^ ± 0.02	1.85^b^ ± 0.09

**Textural parameters of cookie dough**	**Control**	**DS8**	**DS5**	**DS2**
Hardness (*N*)	0.62^a^ ± 0.05	0.49^c^ ± 0.04	0.53^c^ ± 0.02	0.57^b^ ± 0.05
Stickiness (*N*)	0.13^b^ ± 0.02	0.15^a^ ± 0.01	0.15^a^ ± 0.01	0.10^c^ ± 0.03
Strength/Cohesiveness (mm)	0.60^b^ ± 0.02	0.66^a^ ± 0.02	0.67^a^ ± 0.02	0.56^c^ ± 0.04

*Note*: Different superscript lowercase letters in each row show significant differences (*p* < 0.05). WF/PSP8, WF/PSP5, and WF/PSP2 represent the WF‐blends containing 15% of PSP which remained on a sieve with an aperture size of 800, 500, and 212 µm, respectively. DS8, DS5, and DS2 represent the dough samples containing PSP8, PSP5, and PSP2.

### 3.3 Texture Analysis of Cookie Dough

The hardness was reduced as PSP was added to the cookie dough (*p* < 0.05). Moreover, the smallest particle size yielded the hardest dough (Table 3) as observed earlier in short‐type biscuit dough (Sozer et al. 2014) and cracker dough (N. Wang et al. 2016). According to these studies, smaller bran particles were better mixed in the dough matrix, which improved the overall strength. PSP incorporation into the dough increased stickiness and cohesiveness (*p* < 0.05), indicating a sticky dough. Furthermore, the stickiness and cohesiveness of the dough also increased with the increased size of the PSP. However, there were no significant differences between PSP5 and PSP8. Similar results were obtained by Jin et al. (2020), who reported that dough stickiness increased with increasing wheat bran particle size.

### 3.4 Properties of Cookies

Characteristics of cookie samples are given in Table 4. CS2 had substantially higher moisture values (*p* < 0.05) than the other samples. The higher WAC values of PSP2 (Table 1) could be the reason for this situation. Okpala et al. (2013) suggested that flours with high WAC could reduce moisture loss and thus have the potential to retard staling. Lipid content was determined to be similar for all cookies. While the particle size of PSP did not affect the protein and ash content of the cookies, the control sample had substantially (*p* < 0.05) higher protein but lower ash content. PSP‐containing cookies had approximately 86‐ to 100‐fold higher crude fiber content than the control sample, with the highest value observed for CS8 (*p* < 0.05).

**TABLE 4:** Physical and chemical characteristics of cookie samples.

	Control	CS8	CS5	CS2
Moisture (%)	0.75^b^ ± 0.08	0.63^b^ ± 0.12	0.72^b^ ± 0.16	0.95^a^ ± 0.13
Protein (% dw)	5.60^a^ ± 0.13	4.94^b^ ± 0.06	4.91^b^ ± 0.06	4.86^b^ ± 0.06
Lipid (% dw)	17.89 ± 0.78	17.50 ± 0.43	17.31 ± 0.66	17.77 ± 0.33
Ash (% dw)	0.61^b^ ± 0.04	0.67^a^ ± 0.02	0.64^ab^ ± 0.04	0.68^a^ ± 0.02
Crude fiber (% dw)	0.04^c^ ± 0.01	3.57^a^ ± 0.22	3.04^b^ ± 0.09	3.05^b^ ± 0.21
Thickness (*T*) (mm)	6.40^a^ ± 0.29	5.42^c^ ± 0.31	5.48^c^ ± 0.33	5.91^b^ ± 0.38
Diameter (*D*) (mm)	40.22^c^ ±0.62	45.27^a^ ± 0.88	44.99^a^ ± 0.61	43.72^b^ ± 0.60
Spread ratio (*D*/*T*)	6.29^c^ ± 0.31	8.38^a^ ± 0.55	8.24^a^ ± 0.58	7.43^b^ ± 0.51
Hardness (*N*)	37.61^a^ ± 5.48	19.69^c^ ± 3.42	20.41^c^ ± 2.89	27.45^b^ ± 2.73
Fracturability (mm)	38.63^a^ ± 0.34	36.80^c^ ± 0.51	37.26^b^ ± 0.37	38.30^a^ ± 0.49
L*	77.50^a^ ± 1.26	68.21^c^ ± 2.76	69.55^b^ ± 0.90	68.21^c^ ± 0.89
a*	2.77^d^ ± 0.36	4.76^b^ ± 1.59	3.62^c^ ± 0.49	5.29^a^ ± 0.27
b*	25.74^a^ ± 0.69	22.46^b^ ± 1.77	20.16^c^ ± 0.84	22.91^b^ ± 0.41
∆E*	—	10.30 ± 2.80	9.79 ± 0.95	10.04 ± 0.88
Ca (mg/100 g)	23.10^b^ ± 0.47	17.45^d^ ± 0.17	18.19^c^ ± 0.09	31.40^a^ ± 0.16
Cu (mg/100 g)	0.10^a^ ± 0.00	0.06^b^ ± 0.00	0.03^c^ ± 0.01	0.06^b^ ± 0.00
Fe (mg/100 g)	0.03^d^ ± 0.01	0.20^c^ ± 0.02	0.52^b^ ± 0.02	0.65^a^ ± 0.02
K (mg/100 g)	36.72^d^ ± 0.09	118.38^c^ ± 0.03	120.51^b^ ± 0.08	195.16^a^ ± 0.84
Mg (mg/100 g)	3.63^b^ ± 0.02	3.09^d^ ± 0.02	3.49^c^ ± 0.02	4.73^a^ ± 0.09
Mn (mg/100 g)	0.02^c^ ± 0.00	0.03^b^ ± 0.00	0.03^b^ ± 0.00	0.05^a^ ± 0.00
P (mg/100 g)	54.40^b^ ± 4.86	63.51^ab^ ± 6.32	64.36^a^ ± 4.98	69.86^a^ ± 2.89

*Note*: Different superscript lowercase letters in the same row represent significant differences (*p* < 0.05). Cookie samples coded as CS8, CS5, and CS2 contain PSP8, PSP5, and PSP2 which remained on a sieve with an aperture size of 800, 500, and 212 µm, respectively.

Among all tested samples, CS2 demonstrated the highest levels of mineral content (*p* < 0.05) (Table 4). These results suggest that finer PSP may distribute more uniformly within the cookie matrix, contributing more effectively to mineral enrichment due to their increased surface area and better integration. As the particle size increased (PSP5 and PSP8), a declining trend in the mineral contents was observed. This decline may be attributed to the lower dispersibility and potential segregation of coarser particles during the dough formation and baking process (Belorio et al. 2019). These findings are consistent with previous studies conducted by Xiong et al. (2017) and T. Liu et al. (2016), which demonstrated that ingredients rich in coarse fibers, such as bran or shell materials, can hinder gluten development, affect water distribution, and reduce stability of dough. Consequently, this interference impacts the uniform incorporation and availability of nutrients in baked products. However, although higher mineral contents were observed analytically, the bioaccessibility of these minerals during gastrointestinal digestion was not evaluated in this study. Interactions between minerals and dietary fiber or phenolic compounds may limit mineral absorption, and therefore, the observed increases in mineral content may not directly translate into enhanced mineral bioavailability in vivo.

Results showed that PSP incorporation significantly affected the cookies' thickness, diameter, and spread ratio (*p* < 0.05) (Table 4). Compared to the control sample, PSP incorporation decreased the thickness and increased the diameter of the cookies, thereby increasing the spread ratio. The larger the particle size of PSP, the higher the spread ratio. Similarly, Ai et al. (2017) observed lower thickness and higher diameter for the cookies produced with dry bean powders, and they attributed the higher spread ratio values to the coarse particles which could not be hydrated thoroughly and resulted in less cohesive doughs compared to those of fine particles.

The texture analysis results of cookies are shown in Table 4. The findings demonstrated that the hardness and fracturability of cookies made with PSP blends were lower than that of cookies made with refined flour. Moreover, the hardness and fracturability of cookies were impacted by the PSP particle size (*p* < 0.05). Cookies made with the smallest particle size (PSP2) had increased hardness and fracturability. Jia et al. (2020) studied the effect of defatted rice bran addition on biscuit texture and found that the biscuits become softer as a result of a decrease in the gluten network structure. The addition of fiber to the flour diluted the gluten protein and prevented the formation of the gluten network structure. Similarly, Molina et al. (2021) reported that biscuits produced with coarse bran had the lowest textural parameters. In addition, as seen in Table 4, the incorporation of PSP increased the spread ratio of the cookies resulting in decreased hardness and fracturability. The result was similar to the report of Nandeesh et al. (2011), who studied the effect of differently treated wheat bran additives on the physical characteristics of biscuits. Results showed that PSP incorporation significantly affected the L*, a*, b*, and ∆*E* (*p* < 0.05) (Table 4). Compared to the control sample, PSP incorporation decreased the L* and b* values of the cookies, but increased the a* values. However, no significant difference was observed between CS2, CS5, and CS8 in terms of ∆*E*.

As seen in Figure 1b, CS2 was observed to have the highest TPC and AA values (*p* < 0.05). Although the TPC of CS2 was significantly higher than that of CS8 (*p* < 0.05), their AA values were determined to be statistically similar. The amount of other antioxidative substances, such as luteolin, 5,7‐dihydroxychromone, eriodictyol, and 3′,4′,7‐trihydroxyflavanone (Wee et al. 2007) could be higher in PSP8 and thus in CS8. It was also determined by De De Camargo et al. (2014) that peanut skin‐fortified cookies exhibited higher TPC and AA values than the control sample. Nevertheless, it should be noted that TPC and AA measurements reflect the total extractable phenolic content and antioxidant capacity rather than their bioaccessibility. Food matrix structure, interactions, and gastrointestinal metabolism can significantly affect the physiological availability of bioactive compounds. The impact of PSP particle size on the starch fractions in cookies was significant (*p* < 0.05) (Table 5). Although the amount of PSP remained constant, reducing its particle size from 800 to 212 µm resulted in a noticeable decrease in RDS, SDS, and TDS. RS increased significantly in CS2. This suggests that finer PSP particles could enhance the formation or preservation of RS. This result is consistent with previous research by Ng et al. (2017), which demonstrated that dietary fiber‐rich ingredients could disrupt starch structure, reduce digestibility, and improve postprandial glycemic response in biscuits. Furthermore, BeMiller (2011) highlighted that fiber‐rich materials with high water‐holding capacities can compete with starch for water, inhibiting starch gelatinization and enzyme access. These findings emphasize the potential of finely ground dietary fibers to influence starch digestibility and improve the functional properties of baked goods. This is consistent with the higher WAC and OAC values observed for PSP2 (Table 1), which contributed to the reduced starch digestibility in CS2 (Table 5).

**TABLE 5:** Digestible and resistant starch of cookie samples (% dry weight basis).

	Control	CS8	CS5	CS2
RDS	46.20^a^ ± 0.74	45.74^a^ ± 0.13	41.40^b^ ± 0.55	39.51^c^ ± 0.96
SDS	4.51^a^ ± 0.54	4.33^a^ ± 0.14	3.42^a^ ± 0.55	2.10^b^ ± 0.27
TDS	51.18^a^ ± 0.98	49.70^b^ ± 0.25	45.83^c^ ± 0.01	43.62^d^ ± 0.28
RS	0.88^a^ ± 0.01	0.26^c^ ± 0.01	0.32^c^ ± 0.05	0.73^b^ ± 0.03
TS	52.06^a^ ± 0.97	49.95^b^ ± 0.25	46.14^c^ ± 0.04	44.35^d^ ± 0.30

*Note*: Different superscript lowercase letters in the same row represent significant differences (*p* < 0.05). Cookie samples coded as CS8, CS5, and CS2 contain PSP8, PSP5, and PSP2 which remained on a sieve with an aperture size of 800, 500, and 212 µm, respectively.

## 4 Conclusion

This study aimed to valorize PSP, a food waste rich in functional and bioactive compounds, for cookie production and to investigate the effect of its particle size on flour, dough, and cookies characteristics. Particle size significantly influenced the physical, chemical, and textural properties of the cookies, with better results obtained from particles remaining between the 212 and 500 µm sieves. Overall, this study demonstrated that PSP can be valorized as a potential ingredient for developing value‐added food products, particularly in terms of fiber content and antioxidant activity. This study did not focus on the various pretreatments or extraction procedures, nor did it address safety assessments, including potential contaminants, allergenicity, or the stability of bioactive compounds during processing and storage, which were the limitations of the study. Moreover, no bioaccessibility or bioavailability study related to minerals and antioxidant activity was performed. Further studies should focus on the impact of different thermal and non‐thermal processing technologies on this valuable raw material to further improve its functional and technological properties. In addition, toxicological evaluations and shelf‐life stability analyses are also important for validating the possible usage of PSP. Furthermore, the applicability and commercial potential of PSP should be evaluated in various food matrices to gain a broader understanding. In addition, future studies should include more diverse populations to better evaluate the market potential of these products. From a processing perspective, milling, sieving, drying, and mixing operations may pose additional challenges during scale‐up studies, as energy consumption and process efficiency requirements are typically more demanding at the industrial level. Nevertheless, as peanut shells are an abundant agro‐industrial waste, their raw material cost is expected to be relatively low, which may partially offset the additional processing and energy costs associated with industrial‐scale applications.

## Author Contributions

**Ülgen İlknur Konak Alkış**: conceptualization, investigation, writing – original draft, methodology, writing – review and editing, formal analysis, data curation. **Fundagül Erem**: investigation, writing – original draft, methodology, writing – review and editing, formal analysis, data curation. **Nazlı Şahin**: data curation, formal analysis, writing – original draft, writing – review and editing, methodology.

## Conflicts of Interest

The authors declare no conflicts of interest.

## Data Availability

Research data are available in the body of the manuscript.
